# Deep learning-based metastasis detection in patients with lung cancer to enhance reproducibility and reduce workload in brain metastasis screening with MRI: a multi-center study

**DOI:** 10.1186/s40644-024-00669-9

**Published:** 2024-03-01

**Authors:** Yae Won Park, Ji Eun Park, Sung Soo Ahn, Kyunghwa Han, NakYoung Kim, Joo Young Oh, Da Hyun Lee, So Yeon Won, Ilah Shin, Ho Sung Kim, Seung-Koo Lee

**Affiliations:** 1 Department of Radiology and Research Institute of Radiological Science and Center for Clinical Imaging Data Science, Yonsei University College of Medicine, 50-1 Yonsei-ro, Seodaemun-gu, 03722 Seoul, Korea; 2 Department of Radiology and Research Institute of Radiology, University of Ulsan College of Medicine, Asan Medical Center, 43 Olympic-ro 88, Songpa-Gu, 05505 Seoul, Korea; 3 Dynapex, LLC, Seoul, Korea; 4 Department of Radiology, Ajou University Medical Center, Suwon, Korea; 5 Department of Radiology, Samsung Seoul Hospital, Seoul, Korea; 6 Department of Radiology, The Catholic University of Korea, Seoul St. Mary‘s hospital, Seoul, Korea

**Keywords:** Brain metastases, Brain tumors, Deep learning, Magnetic resonance imaging

## Abstract

**Objectives:**

To assess whether a deep learning-based system (DLS) with black-blood imaging for brain metastasis (BM) improves the diagnostic workflow in a multi-center setting.

**Materials and methods:**

In this retrospective study, a DLS was developed in 101 patients and validated on 264 consecutive patients (with lung cancer) having newly developed BM from two tertiary university hospitals, which performed black-blood imaging between January 2020 and April 2021. Four neuroradiologists independently evaluated BM either with segmented masks and BM counts provided (with DLS) or not provided (without DLS) on a clinical trial imaging management system (CTIMS). To assess reading reproducibility, BM count agreement between the readers and the reference standard were calculated using limits of agreement (LoA). Readers’ workload was assessed with reading time, which was automatically measured on CTIMS, and were compared between with and without DLS using linear mixed models considering the imaging center.

**Results:**

In the validation cohort, the detection sensitivity and positive predictive value of the DLS were 90.2% (95% confidence interval [CI]: 88.1–92.2) and 88.2% (95% CI: 85.7–90.4), respectively. The difference between the readers and the reference counts was larger without DLS (LoA: −0.281, 95% CI: −2.888, 2.325) than with DLS (LoA: −0.163, 95% CI: −2.692, 2.367). The reading time was reduced from mean 66.9 s (interquartile range: 43.2–90.6) to 57.3 s (interquartile range: 33.6–81.0) (*P* <.001) in the with DLS group, regardless of the imaging center.

**Conclusion:**

Deep learning-based BM detection and counting with black-blood imaging improved reproducibility and reduced reading time, on multi-center validation.

**Supplementary Information:**

The online version contains supplementary material available at 10.1186/s40644-024-00669-9.

## Introduction

Brain metastases (BMs) are the most frequent intracranial tumors in adults [1]; they occur in 20–40% of patients with systemic cancer and are a major cause of mortality. An early and accurate diagnosis of BMs is crucial for determining treatment strategy and prognosis. A brain MRI with either 3-dimensional gradient echo (3D GRE) or turbo spin echo (3D TSE) is the gold standard for screening patients suspected of having BMs [2]. 3D TSE with black-blood imaging techniques such as improved motion-sensitized driven-equilibrium has shown results superior to those using 3D GRE for detecting small metastases, with double detection rates of BMs less than 5 mm and shorter reading times [3], and is thus considered the “ideal” imaging protocol [2]. Indeed, 3D TSE with black-blood imaging is recommended to replace 3D GRE imaging given its ability to detect small BMs that are missed on 3D GRE imaging [2].

Recently, stand-alone deep learning-based systems (DLSs) have shown detection accuracy for BMs that is comparable to that by radiologists [4–9]. A recent DLS study demonstrated that compared with the 3D GRE alone, adding a 3D TSE with black-blood imaging improves the detection of BMs [6], suggesting that the detection performance of both radiologists and the DLS is higher with the 3D TSE with black-blood imaging. However, actual benefits of DLS-based detection with black-blood imaging in terms of clinical workflow integration remains unclear. Aside from the high accuracy of stand-alone DLS, a significant improvement of radiologists’ performance with the aid of DLS in BM screening should be demonstrated. Reproducibility and workload are essential considerations when assessing the clinically relevant benefits of DLS-based algorithms. The benefits of an interactive DLS should be assessed in terms of overall diagnostic performance as well as reproducibility between radiologists. Moreover, considering that detecting BMs is a tedious and time-consuming task, reducing radiologists’ workload through a DLS is of particular interest. We hypothesized that DLS-based metastasis detection enables automated detection and counting and may enhance diagnostic efficiency in reproducibility and reading time.

Thus, we aimed to assess whether a DLS on a recommended protocol for BM improves the diagnostic workflow in terms of reproducibility and reading time across multiple centers.

## Materials and methods

### Study population

**Fig. 1:**
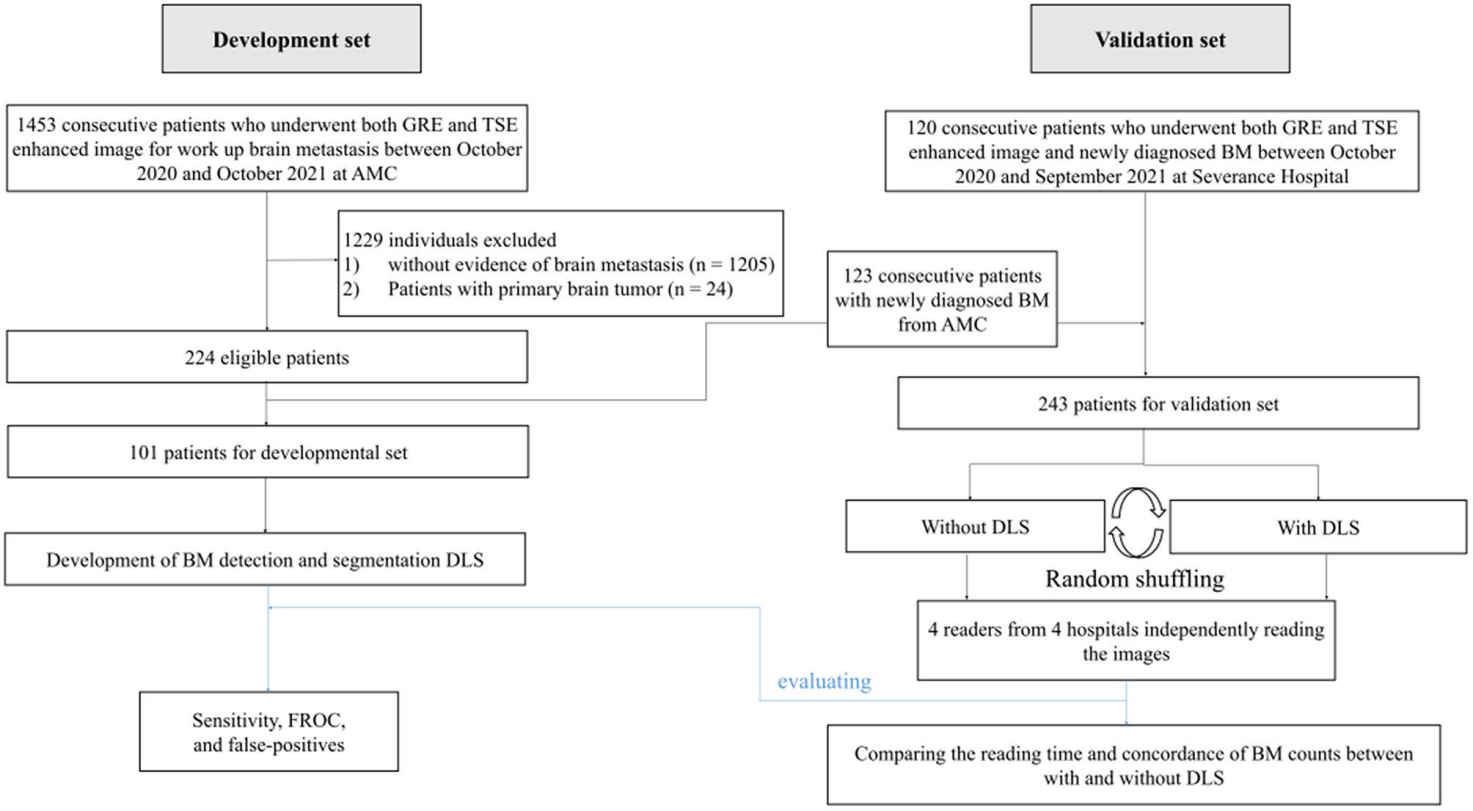
Flow diagram for development and validation with multi-reader evaluation of DLS for detection and segmentation of BM. DLS = deep-learning based system, BM = brain metastasis; GRE = gradient-echo; TSE = turbo spin-echo; FROC = free-response receiver operating characteristic curve analysis

This multi-center retrospective study was approved by the institutional review boards of the participating institutions. Data on the 3D TSE with black-blood imaging have been consecutively obtained since it was implemented in routine clinical practice (Asan Medical Center [Site 1]: 2020; Severance Hospital [Site 2]: 2019). At Site 1, 1453 patients who underwent both a 3D GRE and 3D TSE MRI for metastasis work-up between October 2020 and October 2021 were retrospectively included. Among them, 224 were diagnosed with BMs. Patients were excluded if (1) they had no BM (*n* = 1205) or (2) had other brain tumors (*n* = 24). From the cohort, 101 consecutive patients who underwent both a 3D GRE and 3D TSE MRI were included as a developmental set for the DLS (Fig. 1**)**.

For the validation set, the inclusion criteria were as follows: (1) lung cancer (non-small cell lung cancer) confirmed by pathology, (2) newly developed BMs prior to surgery or radiotherapy, and (3) both a 3D GRE and 3D TSE MRI with at least one follow-up MRI. The exclusion criteria were as follows: (1) diagnosis of a solid tumor other than non-small cell lung cancer and (2) absence of a follow-up study as the reference standard. Between October 2020 and October 2021, 123 and 120 consecutive patients from Sites 1 and 2, respectively, were included as validation sets.

All imaging and clinical data were uploaded and utilized using AiCRO, a clinical trial imaging management system (CTIMS) [10] that meets the current regulatory guidelines and supports computerized system validation. Baseline characteristics included age, sex, primary cancer, imaging acquisition date, and previous local therapy.

### MRI acquisition protocol

Protocols for BMs were in accordance with the recent standardized imaging protocol consensus recommendation [2], including both a 3D GRE and 3D TSE with black-blood (see Table 1 and Supplementary Material S1). Briefly, 3D GRE was MPRAGE (Magnetization Prepared-RApid Gradient Echo) that consists of a non-selective (180°) inversion pulse followed by a collection of rapidly acquired GREs. The imaging parameters for the 3D GRE were as follows: repetition time (TR)/echo time (TE), 9.8/4.6 ms; flip angle, 8°; field of view, 24 cm; section thickness, 1 mm; matrix, 1024 × 1024. The imaging parameters for 3D TSE were TR/TE, 600/28.4 ms; flip angle, 90°; field of view, 24 cm; section thickness, 1 mm; matrix, 240 × 240 or 512 × 512.

**Table 1:** Imaging parameters of 3D GRE and TSE sequence of two centers

	Site 1		Site 2	
Technique	GRE	TSE	GRE	TSE
TR (msec)	9.8	600	5.9–8.6	500
TE (msec)	4.6	28.4	2.8–4.7	28.9–30
Flip angle (degrees)	8	90	8	90
FOV (mm^2^)	240 × 240	240 × 240	240 × 240	240 × 240
Acquisition matrix	512 × 512	512 × 512	240 × 240	240 × 240
Voxel size (mm)	0.5 × 0.5 × 0.5	0.5 × 0.5 × 0.5	1 × 1 × 1	1 × 1 × 1
Slice thickness (mm)	1	1	1	1
Number of excitations	2	1	2	1
Acquisition plane	Sagittal	Sagittal	Sagittal	Sagittal

*Note* GRE = gradient-echo; TSE = turbo spin echo; TR = repetition time; TE = echo time; FOV = field of view

### Image preprocessing and DLS predictions of metastasis

The DLS was trained using a developmental dataset of 101 patients with 864 BMs. The BM segmentation model was implemented using nnU-Net, a 3D U-Net-based method (https://github.com/MIC-DKFZ/nnUNet) [11, 12]. The 3D GRE and 3D TSE image pairs were fed into the model as inputs. A full-resolution 3D model was applied rather than a 2D model or cascade approach (see Supplementary Material S2 and Supplementary Fig. 1). (Source code on https://github.com/jieunp/BM_detection_AI).

### Reference standard for BMs

For reference masks, semi-automatic segmentation of the enhancing tumor region was performed by two researchers (M.S.K. and H.J.K., with 7 and 2 years of experience in radiology, respectively) on co-registered 3D GRE and 3D TSE imaging using MITK software (*www.mitk.org*) [13]. Segmented images were validated by an experienced neuroradiologist (H.S.K., with 18 years of experience in neuro-oncology imaging). It required 15–20 min per patient to make a reference mask. The total number of BMs and ground-truth volumes were recorded separately.

### Image quality check and upload

The processed image masks and 3D GRE and 3D TSE images were de-identified, and the quality was checked by the system manager (A.S., with 5 years of experience in CTIMS) and uploaded to the system (AiCRO). The processed masks were displayed as white masks by applying the maximum values of all images.

### Multi-reader image analysis

Four neuroradiologists from four hospitals (Asan Medical Center, Ajou University Medical Center, Samsung Seoul Hospital, and Seoul St. Mary’s Hospital) with varying degrees of experience (one with > 10 years and three with 5–7 years of experience) were recruited. All readers were blinded to clinical information.

**Fig. 2:**
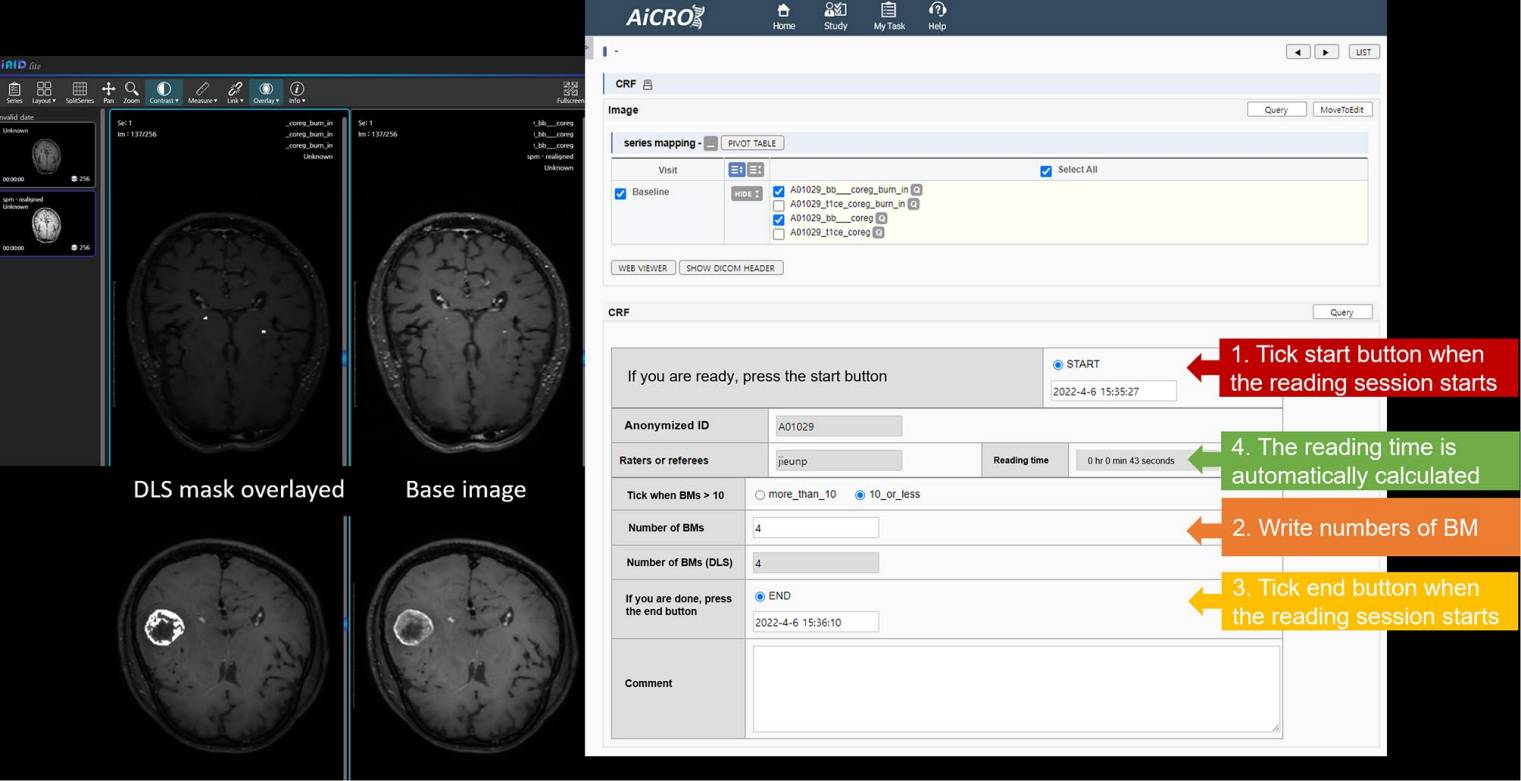
Screenshot of imaging evaluation system using in Clinical Trial Imaging Management System (AiCRO). In with DLS setting, the overlayed DLS masks appears with base image on the left and the base image is displayed on the right with DICOM image format. By clicking the start button, the reading session starts. After reviewing images, the reader writes exact numbers of BM (when BM counts ≤ 10) or clicks on “more than 10” numbers of BM (when BM counts > 10). The reader clicks the end button and the reading time is automatically calculated. DLS = deep learning-based system

The image analyses were conducted over three weeks with case-by-case random shuffling. Images with overlaid processed masks (with DLS) and images without overlayed processed masks (without DLS) were randomly shuffled by the system, and readers evaluated the images sequentially. Before image analysis, the readers were trained in image analysis using 10 sample cases that were not included in the study. Figure 2 illustrates the image evaluation process and Supplementary Video 1 contains a video of the image analysis.

Metastases numbering > 10 per case was labeled “more than 10” BMs; specific numbers of metastases were not counted. This was done for two reasons: (1) assigning a > 10 BMs label is consistent with a recent treatment guideline for BMs [1], which suggests that whole-brain radiotherapy or systematic chemotherapy should be considered for patients with more than 10 metastases and (2) in clinical practice, radiologists often report the number of BMs as “multiple” or “numerous” when > 10 are found, and we aimed for the workload to account for this real-world situation.

### Statistical analysis

1) Detection performance of the DLS: Findings were considered true-positive when at least one voxel was overlapped with the ground-truth volume. Meanwhile, findings were considered false-positive (FP) when no voxel was overlapped with the ground truth volume. The sensitivity, positive predictive value (PPVs), and number of FPs per patient were calculated. Generalized estimating equation (GEE) modeling was used to calculate 95% confidence intervals (CIs) to account for clustering of multiple measurements per case. The free-response receiver operating characteristic (FROC) curve was also calculated.

2) Reproducibility among readers: The number of metastases recorded by the readers was compared for reproducibility. Scatter plots of the with and without DLS groups were drawn. The concordance correlation coefficient (CCC) was used as a reproducibility index to quantify agreement between the assessments. A Bland-Altman analysis with 95% limits of agreement (LoA) assessed agreement between the readers’ counts and reference standard counts [14, 15].

3) Workload assessment: The difference in reading time between the with and without DLS groups was calculated for each reader and compared. To account for repeated assessments of the given cases, we used linear mixed models and readers were incorporated as a random effect. Subgroup analysis was also performed for the workload associated with counting the specific number of BMs (≤ 10) and assigning a > 10 BMs label.

Statistical analyses were performed by an expert biostatistician (K.H., with 15 years of experience) using R software (version 4.1.2) with the packages ‘lmerTest’ and ‘DescTools’. Statistical significance was set at *P* value < 0.05.

### Data availability

The datasets generated or analyzed during the study are available from the corresponding author on reasonable request.

## Results

### Patient demographics

Table 2 summarizes the clinical characteristics of the 101, 123, and 120 patients in the developmental set, Site 1 validation set, and Site 2 validation set, respectively. There were no significant differences in age or sex between the two validation sets.

**Table 2:** Patient characteristics and information on brain metastases

	Developmental set	Validation set		
	Site 1	Site 1	Site 2	*P-value*
Number of patients	101	123	120	
Age	61.4 ± 9.2	65.0 ± 9.7	62.6 ± 11.9	0.08
Male Sex	60 (59.4)	68 (55.3)	72 (60)	0.42
BM Number	864	2078	832	
Average number of BM for each patient	8.6 ± 9.6	8.7 ± 12.2	7.0 ± 6.6	**0.015**
Patients with > 10 numbers of BM	20 (19.8)	32 (26.0)	19 (16.8)	0.050
Volume and size (mm^3^) of BM				
Mean ± SD (volume, mm^3^)	204.9 ± 811.8	211.5 ± 1406.9	345.7 ± 1569.4	**0.024**
Mean ± SD (diameter, mm)	4.69 ± 3.57	4.53 ± 3.52	4.45 ± 4.55	0.625
BM numbers less than 5 mm in diameter	689 (79.5)	1697 (81.6)	619 (75.6)	0.378
Primary tumor types				0.587
Lung adenocarcinoma	85 (84.2)	115 (93.5)	110 (91.6)	
Lung squamous cell carcinoma	5 (4.9)	8 (6.5)	10 (8.3)	
Lung other types of cancer	2 (1.9)			
Breast cancer	5 (4.9)			
Colon cancer	2 (1.9)			
Renal cancer	4 (1.9)			

Data are expressed as the mean ± standard deviation or numbers with percentages in parentheses. *P* value indicates statistical significance between two hospitals in the validation set

*Abbreviation* BM = brain metastasis; SD = standard deviation

In the developmental and validation sets, the total number of BMs was 864 (developmental set), 2,078 (Site 1), and 832 (Site 2). The mean number of BMs per patient was 8.6 ± 9.6 (developmental set), 8.7 ± 12.2 (Site 1), and 7.0 ± 6.6 (Site 2). The mean diameter of the metastases was 4.69 ± 3.57 mm (developmental set), 4.53 ± 3.52 (Site 1), and 4.45 ± 4.55 (Site 2) and the proportion of BMs smaller than 5 mm was 79.5% (developmental set), 81.6% (Site 1), and 75.6% (Site 2). The distribution and size of the BMs across patients are shown in Supplementary Fig. 2.

### Detection performance of the DLS

**Fig. 3:**
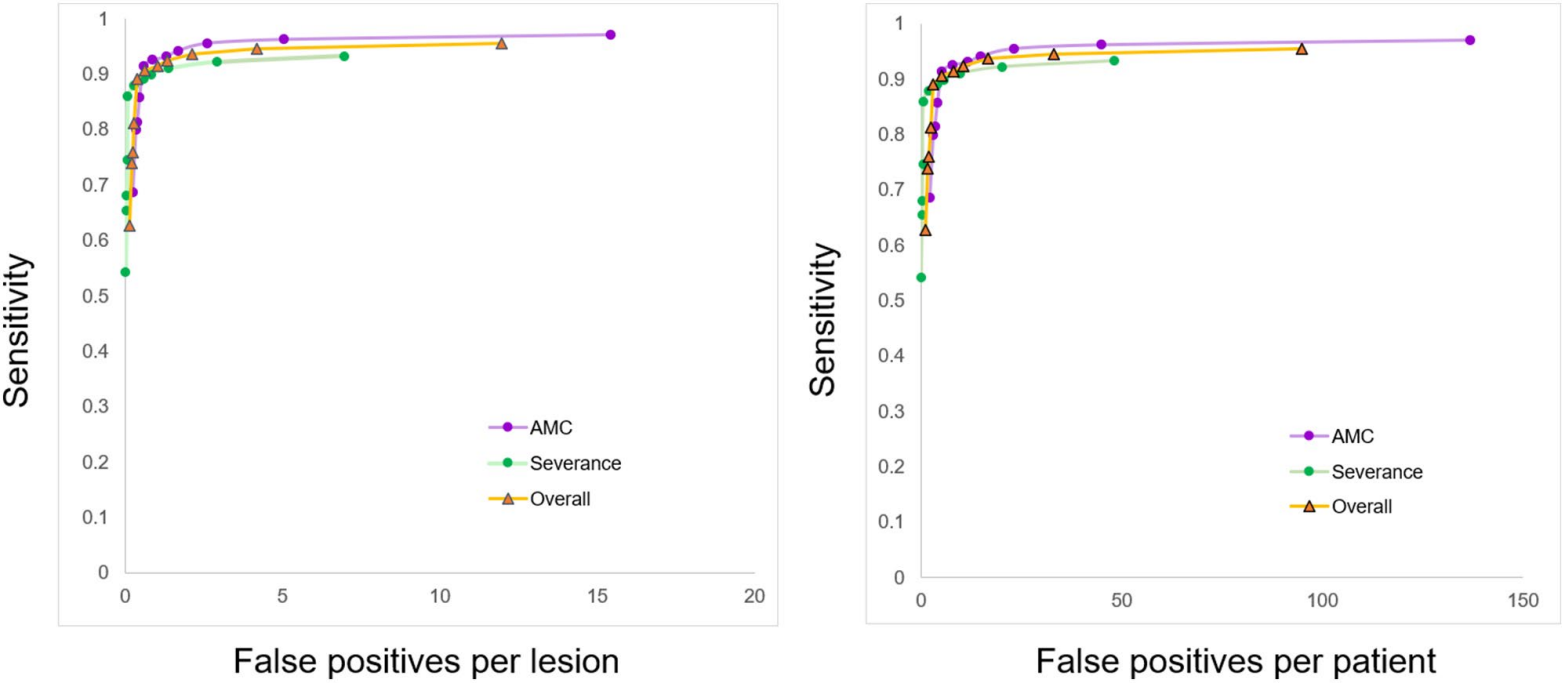
The performance of DLS for BM. The lesion-based and patient-based FROCs of DLS is shown. FROC = free-response receiver operating characteristic

In the developmental set, the DLS showed a sensitivity of 87.7% (758/864, 95% CI: 84.0–90.5) and positive predictive value of 89.0% (758/840, 95% CI: 84.7–92.6). In the validation set, the DLS showed an overall detection sensitivity of 90.2% (2625/2910, 95% CI: 88.1–92.2) and PPV of 88.2% (2624/2974, 95% CI: 85.6–90.4). Figure 3 illustrates the FROC curve for BMs in the validation set. The number of FPs per patient was 1.44 (350 FPs from 243 patients).

Table 3 summarizes the sensitivity, PPV, and FPs per patient for each hospital. Supplementary Fig. 3 shows the FROC for the developmental set.

**Table 3:** Performance of deep learning system in the validation set per each hospital

Performance	Site 1	Site 2
Sensitivity	90.9% [1890/2078] (88.4–93.4%)	88.3% [735/832] (85.9–91.6%)
PPV	87.9% [1890/2148] (85.3–90.1)	88.9% [734/826] (86.1–91.0)
FPs per patient	2.09 [258/123]	0.77 [92/120]

FP = false-positive; PPV = positive predictive value

### Reproducibility among readers

**Fig. 4:**
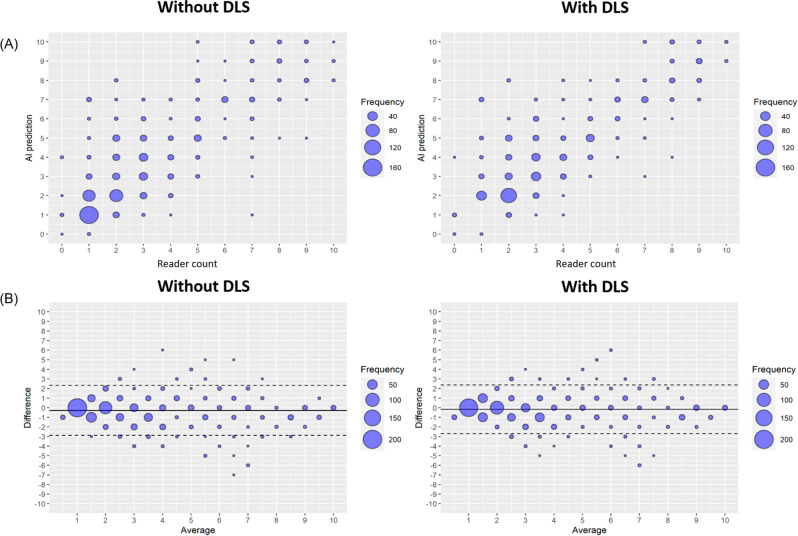
Distribution of numbers of BM equal or less than 10 counts. **(A)** The scatter plots of number of BM counts in “assessment without DLS” and “assessment with DLS” setting. The readers report less dispersed numbers of BMs when assisted with DLS. **(B)** Bland-Altman plots with 95% limits of agreement (LoA) exhibiting differences between reader counts and reference standard. The “assessment without DLS” shows wider LoA compared with “assessment with DLS”. DLS = deep learning-based system

Table 4 summarizes the results of reproducibility among readers. The CCC for the number of BMs detected between the readers and the reference standard was higher with the DLS (0.918, 95% CI: 0.901–0.933) than without the DLS (0.897, 95% CI: 0.876–0.915). Scatterplots of the BM counts in the with and without DLS groups are shown in Fig. 4A. The distribution was less dispersed when the readers were assisted with the DLS, indicating that agreement among readers increased when the DLS was used.

**Table 4:** Reproducibility between the readers’ counts and reference standard counts in the without DLS and with DLS setting

	Without DLS	With DLS
CCC	0.897 (0.876, 0.915)	0.918 (0.901, 0.933)
LoA between readers’ counts and reference standard counts	−0.281 (− 2.888, 2.325)	−0.163 (− 2.692, 2.367)

Data are expressed as means with 95% confidence intervals in parentheses. CCC was calculated with BM numbers equal or less than 10

CCC = concordance correlance coefficient; LoA = limits of agreement

The Bland-Altman plot for the difference between the readers’ counts and the reference counts is shown in Fig. 4B. This difference was larger in the without DLS group (LoA: −0.281, 95% CI: −2.888, 2.325) than in the with DLS group (LoA: −0.163, 95% CI: −2.692, 2.367).

### Workload assessment

Table 5 gives the workload assessment results. The mean reading time was 66.9 s without the DLS and 57.3 s with the DLS. Thus, the DLS significantly reduced the reading time by 9.6 s (95% CI: 7.3–12.0) (*P* <.001).

**Table 5:** Comparison of workload in reading BM between with DLS and without DLS setting

Time [seconds]	Without DLS	With DLS	Difference	*P*-value
**All**	66.9 (43.2, 90.6)	57.3 (33.6, 81.0)	9.6 (7.3, 12.0)	< 0.001
**BM numbers**				
≤ 10	72.5 (45.6, 99.4)	63.5 (36.6, 90.4)	9.1 (6.4, 11.6)	< 0.001
> 10	56.4 (43.2, 69.5)	43.6 (30.5, 56.8)	12.7 (8.8, 16.6)	< 0.001
**Imaging Center**				
Site 1	71.5 (48.2, 94.7)	57.5 (34.3, 80.8)	14.0 (10.7, 17.2)	< 0.0001
Site 2	62.3 (39.0, 85.5)	57.05 (33.8, 80.3)	5.2 (1.9, 8.5)	0.0018
**Imaging center & BM numbers**				
≤ 10				
Site 1	79.9 (53.5, 106.3)	66.1 (39.7, 92.5)	13.8 (10.0, 17.6)	< 0.0001
Site 2	65.3 (38.9, 91.8)	60.7 (34.2, 87.1)	4.7 (1.1, 8.3)	0.011
> 10				
Site 1	55.4 (41.1, 69.6)	40.9 (26.6, 55.1)	14.5 (9.6, 19.5)	< 0.0001
Site 2	56.8 (40.5, 73.1)	47.2 (30.8, 63.6)	9.6 (3.2, 16.0)	0.004

*Note* The unit number is seconds. Data are expressed as means with standard deviations in parentheses

*Abbreviation* DLS = deep learning-based system

The reading time was longer when the specific number of BMs was counted instead of assigning the > 10 BMs label for either with or without the DLS. The DLS greatly reduced the reading time for either counting the specific number of BMs (difference 9.1s, *P* <.001) or assigning the > 10 BMs label (difference of 12.7s, *P* <.001). The effect of DLS for reducing the reading time was more pronounced for the > 10 BMs label than counting the specific number of BMs.

The DLS significantly reduced the reading time regardless of the imaging center (difference of 14.0 and 5.2s in Sites 1 and 2, respectively). The effect on reading time was significant in all centers for either counting a specific number of BMs or assigning the > 10 BMs label (Site 1, overall: *P* <.0001; Site 2, overall: *P* =.0018; Site 1, equal or less than 10 BMs: *P* <.0001; Site 2, equal or less than 10 BMs: *P* =.011; Site 1, > 10 BMs: *P* <.0001, Site 2, > 10 BMs: *P* =.004).

## Discussion

To date, reproducible and quantitative imaging endpoints for BM have not been available for DLS owing to high FP detections from 3D GRE. In this study, the clinical value of DLS with black-blood imaging for the detection and counting of BM was demonstrated. The inter-rater agreement among radiologists and the agreement between the readers’ counts and reference standard counts increased with DLS. The readers’ reading time was significantly reduced with DLS, regardless of number of BM or imaging centers, demonstrating workload reduction. The DLS showed a sufficient detection performance with a sensitivity and PPV of 90.2% and 88.2%, respectively. To our knowledge, the present study is the first to address real benefits in terms of reproducibility and workload of DLS with black-blood imaging, which is the recommended protocol for clinical trials of BMs [2].

Variation in inter-reader performance is a well-known problem in BM detection. A recent study found BM detection sensitivity to vary from 66.4 to 88.1% among radiologists with different levels of experience [4]. Since software is robust to human variation, DLS may contribute to reducing variability in radiologists’ diagnostic performance [16]. Our results show that inter-reader agreement in terms of CCC increases with DLS compared to that without DLS (from 0.896 to 0.917), suggesting that assistance of DLS allows a more stable and reproducible assessment. These findings are supported by decreased LoA with DLS compared to without the DLS (from − 0.281 to − 0.163), indicating greater agreement between reader and reference standard with DLS. Increased reproducibility will increase the reliability of radiologists’ interpretation, regardless of their experience level.

The efficiency of BM detection, particularly in terms of workload, is another important aspect of DLS implementation. Studies have shown a decrease in the reading time by 40 to 85s with DLS [4, 17]; however, since these studies used 3D GRE imaging, the reading times for both with and without DLS groups were remarkably longer than those in our DLS study with black-blood imaging. Specifically, the reading time ranged from 72 to 85s in the with DLS group and 114 to 140s in the without DLS group with 3D GRE imaging [4, 17], while in our study, shorter reading times of 57.3 and 66.9s in with and without DLS groups, respectively, were noted. A previous study has already shown a significant decrease in reading time by up to 30 s with 3D TSE compared to 3D GRE imaging, while the reading time with 3D TSE ranged from 45.5 to 53.7s [3], and our results further demonstrate that DLS with black-blood imaging is even more efficient than DLS without black-blood imaging.

The robustness and clinical utility of DLS for detecting BMs need to be challenged to fully understand its strengths and limitations. Majority of previous studies applying deep learning for BMs were single-center studies [4, 6–8, 18–20], which critically limits the generalizability of the DLS. Moreover, previous studies have only reported the stand-alone performance of DLSs [6–9, 18–20], which lacks clinical feasibility as it is currently ethically and legally impossible for DLS to be considered an independent neuroimaging reader. Thus, the imminent clinical scenario of implementation of DLS in BM detection is as an assistance of radiologist rather than as a replacement.

In our study, 3D TSE with black-blood imaging was the reference standard for BM detection. Our DLS showed a high sensitivity of 90.2% for BM detection, which is higher than that of the majority of previous DLS studies (range, 81–91%), which used only 3D GRE images [4, 8, 19, 20]. A recent DLS study using 3D GRE imaging showed that with DLS, radiologists’ detection sensitivity improved from 92.7 to 95.0% [17], similar to our findings. However, these results should be interpreted with caution since there is a substantially different proportion of small BMs in these studies; 42.7% of the ground-truth lesions in our study were < 3 mm, while 14.2% of the lesions in the DLS study using 3D GRE were < 3 mm [17]. The pooled detection sensitivity of radiologists has been reported to be higher with 3D TSE than with 3D GRE images (89.2% vs. 81.6%) [21], which suggests the possibility that DLS studies using 3D GRE imaging may have incomplete ground-truth masks with missed lesions. The use of 3D TSE with black-blood imaging in our study may have contributed to the increased detection of small BMs for ground-truth masks, which were sensitively detected in DLS.

FP per patient is the most commonly used metric in articles on BM detection with deep learning [8, 9]. Of note, FPs in Site 1 was 2.09 (258/123), which was higher than that in Site 2 0.77 (92/120). We speculate there are two reasons: first, the average number of BMs for each patient was higher in Site 1 (8.7 per patient) compared with Site 2 (7.0), which simply increased the number of FP cases. Second, there were several patients with extremely large number of BMs (33–65 BMs) in Site 1, which contributed to increase the number of FP cases per patient. This increased number of ‘per patient’ calculation is supported by the fact that sensitivity (true positive cases/disease positive cases) and PPV (true positive cases/test positive cases) between two sites are similar.

Our study had several limitations. First, our model was trained and tested on lung cancer patients with BMs. Because the incidence of BMs is lower when screening, with a reported rate of 26.8% in lung cancer [22], a prospective study including patients without BMs is warranted. Second, our DLS implemented 3D black-blood imaging, which may not be available in all scanners. Third, the readers counted the numbers of BMs and the reading time was automatically reported, which simulated as a real workflow in radiology, and did not draw region of interest (ROI) for each metastasis. DLS as a second reader was evaluated in terms of workflow efficiency and not from diagnostic efficacy. Further prospective study combining diagnostic efficacy and workflow efficiency can be designed for guiding stereotactic radiosurgery reflecting the actual clinical workflow.

## Conclusion

In conclusion, deep learning-based metastasis detection and counting with black-blood imaging improved reproducibility and enhanced diagnostic efficiency through a reduction in reading time, with multi-center validation.

### Electronic supplementary material

Below is the link to the electronic supplementary material.

**Supplementary Material 1: S1.** MRI acquisition protocol. **S2.** Image preprocessing and DLS prediction of metastasis

**Supplementary Material 2: Supplementary Figure 1.** Network architecture of the deep learning system for detection and count of brain metastasis. Since the input image size varies for each case, several patches are generated using a sliding window approach. Model prediction results for each patch overlap by half of the size of a patch and are aggregated to generate the final lesion mask

**Supplementary Material 3: Supplementary Video 1.** A representative video of the imaging evaluation process using in Clinical Trial Imaging Management System (AICRO) in with AI setting

**Supplementary Material 4: Supplementary Figure 2.** Distributions of brain metastasis sizes and numbers in the developmental and validation sets. The data from two hospitals are colored in purple (Site 1) and green (Site 2) in the validation set

**Supplementary Material 5: Supplementary Figure 3.** The performance of DLS for BM in the developmental set

## Data Availability

The datasets generated or analyzed during the study are available from the corresponding author on reasonable request.

## Abbreviations

3D GRE: 3-dimensional gradient echo
3D TSE: 3-dimensional turbo spin echo
BM: brain metastasis
CCC: concordance correlation coefficient
CI: confidence interval
DLS: deep learning-based system
FP: false-positive
FROC: free-response receiver operating characteristic
GEE: generalized estimated equation
LoA: limits of agreement
PPV: positive predictive value
