# Correction: Deep learning-based metastasis detection in patients with lung cancer to enhance reproducibility and reduce workload in brain metastasis screening with MRI: a multi-center study

**DOI:** 10.1186/s40644-024-00688-6

**Published:** 2024-03-21

**Authors:** Yae Won Park, Ji Eun Park, Sung Soo Ahn, Kyunghwa Han, NakYoung Kim, Joo Young Oh, Da Hyun Lee, So Yeon Won, Ilah Shin, Ho Sung Kim, Seung-Koo Lee

**Affiliations:** 1 Department of Radiology and Research Institute of Radiological Science and Center for Clinical Imaging Data Science, Yonsei University College of Medicine, 50-1 Yonsei-ro, Seodaemun-gu, 03722 Seoul, Korea; 2 Department of Radiology and Research Institute of Radiology, University of Ulsan College of Medicine, Asan Medical Center, 43 Olympic-ro 88, Songpa-Gu, 3Dynapex, LLC, 05505 Seoul, Korea; 3 Dynapex, LLC, Seoul, Korea; 4 Department of Radiology, Ajou University Medical Center, Suwon, Korea; 5 Department of Radiology, Samsung Seoul Hospital, Seoul, Korea; 6 Department of Radiology, The Catholic University of Korea, Seoul St. Mary‘s hospital, Seoul, Korea

Following publication of the original article [1], we were notified that the description and order of the supplementary files was incorrect. This has now been rectified.

The original article has been corrected.
