# Supplementary material for: Deep learning-based metastasis detection in patients with lung cancer to enhance reproducibility and reduce workload in brain metastasis screening with MRI: a multi-center study
Source: Cancer Imaging. 2024 Mar 1;24:32. doi: 10.1186/s40644-024-00669-9 (PMC10905821; doi:10.1186/s40644-024-00669-9)
Supplement: Supplementary file 1 — Supplementary Material 1: S1. MRI acquisition protocol. S2. Image preprocessing and DLS prediction of metastasis [file 40644_2024_669_MOESM1_ESM.docx]

**Supplementary Material 1.**

**Supplementary Material S1. MRI acquisition protocol**

Site 1: 3D GRE sequence was MPRAGE (Magnetization Prepared-RApid Gradient Echo) that consists of a non-selective (180°) inversion pulse followed by a collection of rapidly acquired GREs. The imaging parameters for the GRE-T1WI were as follows: repetition time (TR)/echo time (TE), 9.8/4.6 ms; flip angle, 8°; field of view, 24 cm; section thickness, 1 mm; matrix, 1024 × 1024; and acquisition time, 2 minutes 58 s. The parameters for the fast spin-echo sequence were as follows: TR/TE, 600/28.4 ms; flip angle, 90°; field of view, 24 cm; section thickness, 1 mm; matrix, 512 × 512; and acquisition time, 3 minutes 33 s. The improved motion-sensitized driven-equilibrium pre-pulse consisted of one 90° excitation pulse, two 180° refocusing pulses, and one 90° excitation pulse with motion-sensitized gradients between radiofrequency pulses. The duration between the two 90° pulses (TEprep) was 28.3 ms, and the flow velocity encoding for gradient pulses was 3 cm/s.

Site 2: The parameters for the fast spin-echo sequence were as follows: TR/TE, 600/28.4 ms; flip angle, 90°; field of view, 24 cm; section thickness, 1 mm; matrix, 240 × 240; and acquisition time, 3 minutes 23 s. The improved motion-sensitized driven-equilibrium pre-pulse consisted of one 90° excitation pulse, two 180° refocusing pulses, and one 90° excitation pulse with motion-sensitized gradients between radiofrequency pulses. The duration between the two 90° pulses (TEprep) was 28.3 ms, and the flow velocity encoding for gradient pulses was 1.3 cm/s. The imaging parameters for the GRE-T1WI sequence were as follows: repetition time (TR)/echo time (TE), 5.9-8.6/2.8-4.7 ms; flip angle, 8°; field of view, 24 cm; section thickness, 1 mm; matrix, 240 × 240; and acquisition time, 3 minutes 2 s.

**Supplementary Material S2. Image preprocessing and DLS prediction of metastasis**

The DLS network was trained with a separate developmental dataset of 101 patients with 864 brain metastases, who were not included in the clinical cohort. Paired 3D GRE and 3D TSE imaging data were co-registered using rigid transformations with six degrees of freedom in SPM (version 12, www.fil.ion.ucl.ac.uk/spm/). Skull stripping was performed using an algorithm (https://github.com/MIC-DKFZ/HD-BET) optimized for heterogeneous MRI data with diverse pathology or post-treatment changes.^1^ The lesion segmentation model was implemented using nnU-Net, a 3D U-Net-based method (https://github.com/MIC-DKFZ/nnUNet)^2,3^ in the PyTorch package version 1.1 in Python 3.7 (www.python.org). The nnU-Net has basically the same neural net design as U-Net and concentrate their effort to pre/post data processing and hyper-parameters for more practical use and better performance.^3^ In our model training, a 3D GRE and 3D TSE image pair are fed into the model as input. Then, the nnU-Net automatically determines hyperparameters for model training in consideration of the core characteristics (called ‘dataset fingerprint’) of the dataset, such as the class ratios, image size, or voxel spacing information. It was easier to obtain a holistic working deep learning model since it covers from data augmentation to patch generation and stitching patches to get the final prediction result.

A full-resolution 3D model was applied rather than a 2D model or cascade approach. A 3D model was expected to show better performance in metastasis because the shape of the lesion was simple with small size and there could be multiple lesions in a single image. The data were augmented with rotation, gamma, scaling, elastic deformation, and mirror transforms. The model training details includes: Dice and cross-entropy loss function, Adam optimizer^4^ with an initial learning rate of 3 × 10^−4^ and a weight decay of to 3 × 10^−5^, batch size 2, and patch size 128 × 128 × 112. Model training was conducted with NVIDIA TITAN RTX 24 GB GPU with CUDA version 10.0, and the model performance converged after 507 epochs in 2.5 days.

**References**

1 Isensee, F. *et al.* Automated brain extraction of multisequence MRI using artificial neural networks. *Hum Brain Mapp* **40**, 4952-4964 (2019). <https://doi.org/10.1002/hbm.24750>

2 Isensee, F. *et al.* Automated brain extraction of multisequence MRI using artificial neural networks. *J Time Ser Anal* **40**, 4952-4964 (2019). <https://doi.org/10.1002/hbm.24750>

3 Isensee, F., Jaeger, P. F., Kohl, S. A. A., Petersen, J. & Maier-Hein, K. H. nnU-Net: a self-configuring method for deep learning-based biomedical image segmentation. *Nat Methods* **18**, 203-211 (2021). <https://doi.org/10.1038/s41592-020-01008-z>

4 Kingma, D. P. & Ba, J. Adam: A method for stochastic optimization. *arXiv preprint arXiv:1412.6980* (2014).
