# Supplementary figures and images for: Deep learning-based metastasis detection in patients with lung cancer to enhance reproducibility and reduce workload in brain metastasis screening with MRI: a multi-center study
Source: Cancer Imaging. 2024 Mar 1;24:32. doi: 10.1186/s40644-024-00669-9 (PMC10905821; doi:10.1186/s40644-024-00669-9)

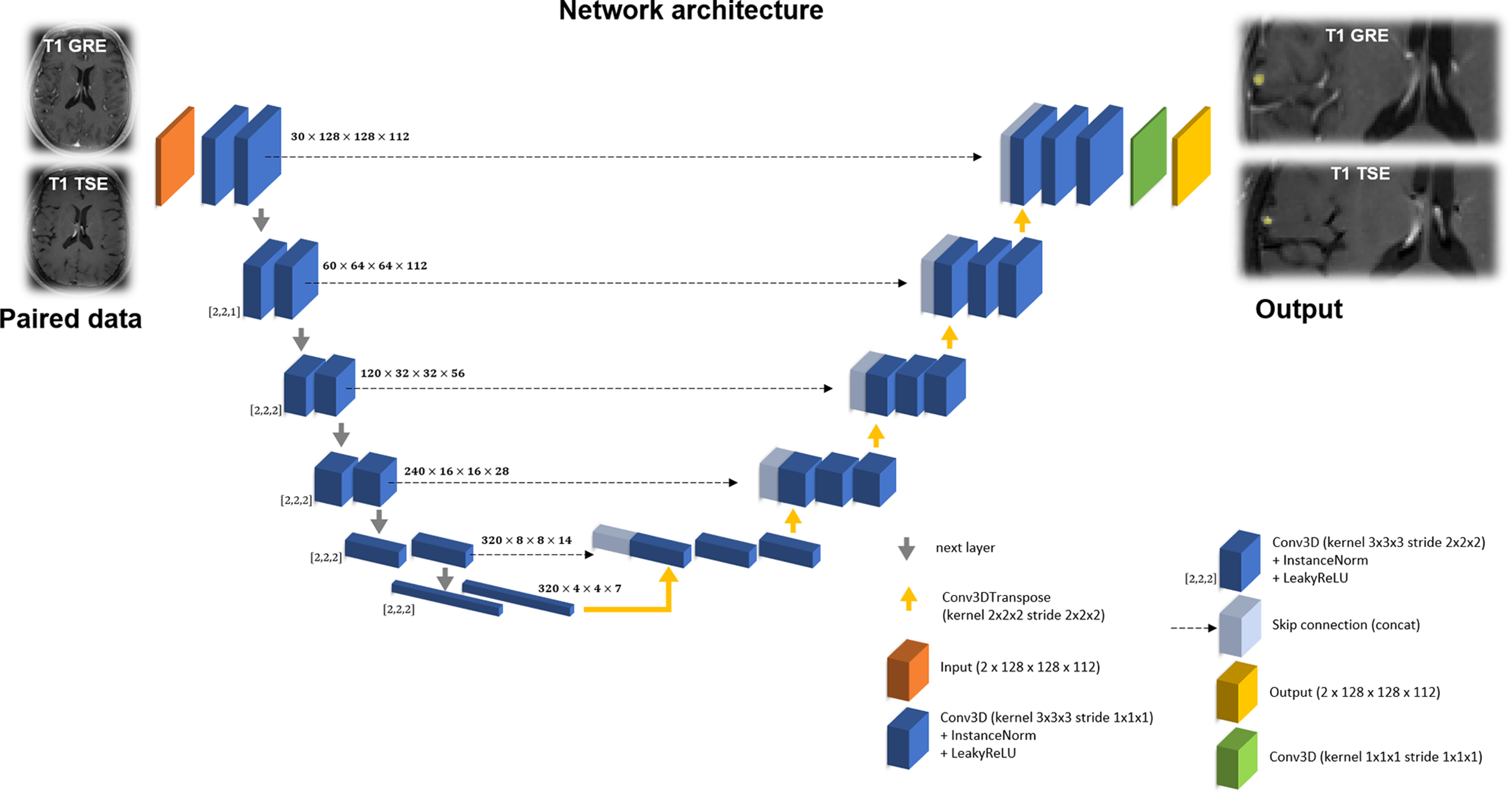

Supplement: Supplementary file 2 — Supplementary Material 2: Supplementary Figure 1. Network architecture of the deep learning system for detection and count of brain metastasis. Since the input image size varies for each case, several patches are generated using a sliding window approach. Model prediction results for each patch overlap by half of the size of a patch and are aggregated to generate the final lesion mask [file 40644_2024_669_MOESM2_ESM.tif]

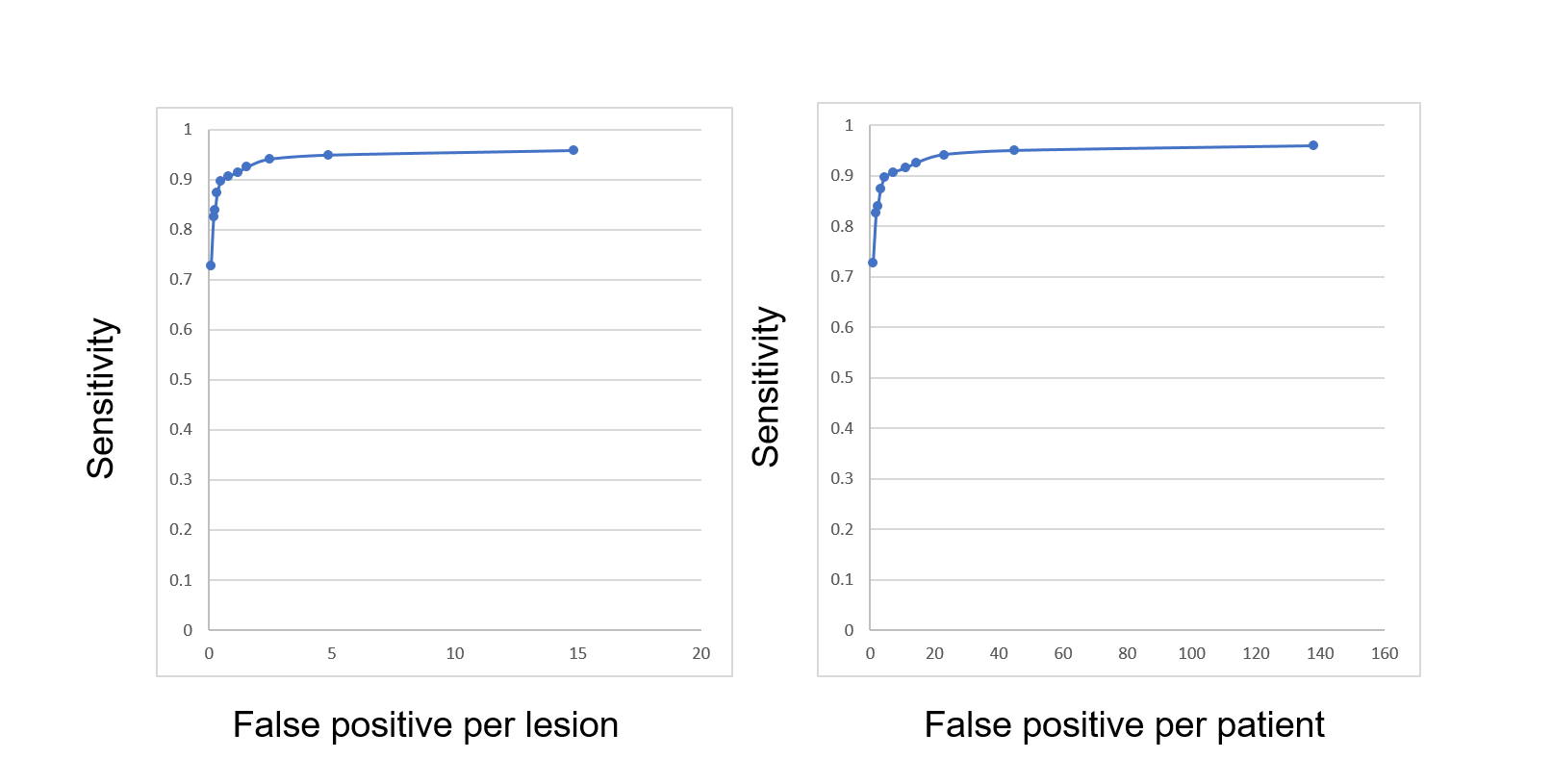

Supplement: Supplementary file 5 — Supplementary Material 5: Supplementary Figure 3. The performance of DLS for BM in the developmental set [file 40644_2024_669_MOESM5_ESM.tif]
